# Light People: Academician Junhao Chu

**DOI:** 10.1038/s41377-023-01147-w

**Published:** 2023-05-05

**Authors:** Hui Wang, Rongjun Zhang, Cun Yu

**Affiliations:** 1 Changchun Institute of Optics, Fine Mechanics and Physics, Chinese Academy of Sciences, 3888 Dong Nan Hu Road, 130033 Changchun, China; 2 Fudan University, 220 Handan Road, Yangpu District, 200433 Shanghai, China

**Keywords:** Photonic devices, Imaging and sensing

## Abstract

The industrial revolutions of steam power, electric power and digital power have been three key steps in the development of science and technology. Now, the fourth industrial revolution has quietly begun, a revolution which will combine the powers of modern technologies such as the Internet, industrial digitalization and virtual reality to trigger a major change of science and technology, and sensor technology is of vital importance to this process.

A famous physicist specialized in infrared and semiconductors, our featured guest not only discovered the intrinsic absorption spectra of the optical transition between narrow gap semiconductor mercury cadmium telluride bands, but also developed the theory of the band structure of mercury cadmium telluride and the theory of optical transition, put forward a series of expressions such as the gap width of mercury cadmium telluride bands, making outstanding contributions.

Born into a scholarly family, he inherited a love for knowledge, especially physics and enjoyed all the challenges it presented. In research, he believes that technological development should be guided by the laws of physics. As a teacher, he asks his students to focus on the depth and breadth of learning. In life, he is famed for being easygoing, modest, well-mannered and meticulous.

He is Academician Junhao Chu of the Shanghai Institute of Technical Physics (SITP), Chinese Academy of Sciences (CAS). Please follow Light People and discover what challenges Prof. Chu had to overcome in the study of mercury cadmium telluride.

## Abstract (web summary)

Academician Junhao Chu, specializing in infrared and semiconductors, makes outstanding contributions by putting forward a series of expressions such as the gap width of mercury cadmium telluride bands, etc.

## Graphical abstract

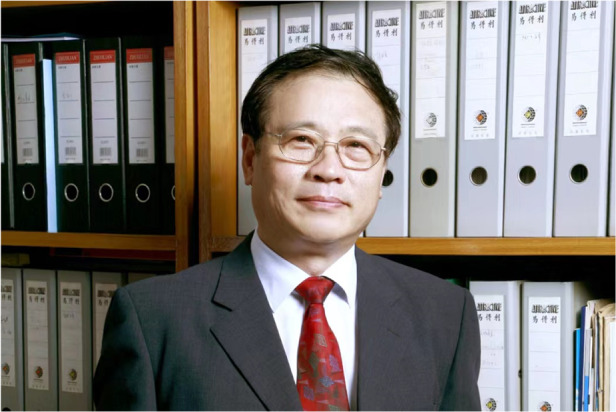


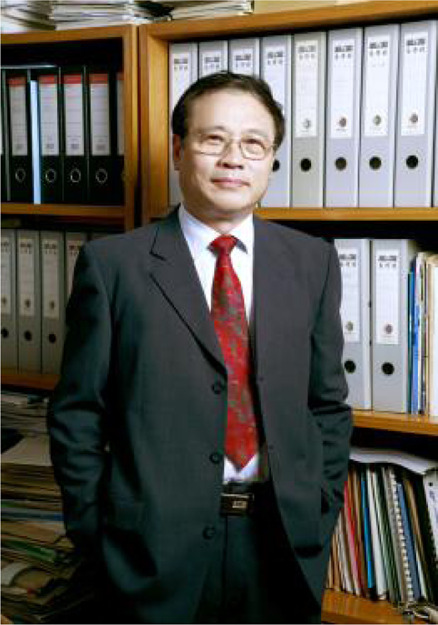


Professor Junhao Chu, an infrared physicist and expert in semiconductor physics and devices, is an academician of CAS, a researcher of SITP, and Dean of the Institute of Opto-Electronics at Fudan University. Prof. Chu graduated from the Physics Department of Shanghai Normal University in 1966. In 1981 and 1984, he received his Master’s and Doctor’s degrees from SITP. From 1986 to 1988, he did research work on two-dimensional electronic gas in semiconductors at the Physics Department of Technical University of Munich, Germany, sponsored by the Humboldt Foundation. 1993–2002, he was director of the State Key Laboratory of Infrared Physics, SITP. He was elected academician of CAS in 2005. Prof. Chu has long been engaged in the research of infrared optoelectronic materials and devices, and has conducted material physics and device research on narrow gap semiconductor mercury cadmium telluride (HgCdTe) and ferroelectric thin films used for infrared detectors.

**1. Could you briefly introduce your research direction and content?**

Academician Chu: My research is mainly on infrared optoelectronic materials and devices, which involves semiconductor physics, condensed matter physics, optics and electronics. Narrow band gap semiconductor is a very important branch of semiconductor physics. Germanium and silicon are materials used to make traditional semiconductors, but there are semiconductors with a very narrow band gap width, such as HgCdTe, InSb and so on. Narrow gap semiconductors and other related materials that respond in infrared band are my specific research field.

**2. You have made many achievements in the research of infrared optoelectronic materials and devices, including obtaining the most direct physical significance of mercury cadmium telluride band gap width, composition and temperature relationship, known internationally as the CXT (Chu-Xu-Tang) formula; established a theoretical model to study the structure of narrow gap semiconductor MIS devices two-dimensional electron gas band structure, etc. Which achievement are you most proud of so far?**

Academician Chu: In the CXT formula, C is Junhao Chu, X is Shiqiu Xu, and T is Dingyuan Tang. I received a lot of help and support when I was doing this research. The materials group provided me with a large number of samples of mercury cadmium telluride. Jiaxiong Fang gave me access to 4.2K–300K variable Dewars bottles imported from the UK. Shiqiu Xu let me use his PE 983 spectrometer and all the equipment in the infrared spectrum laboratory.

So far, I am most satisfied with the following results:

The first is the achievement of narrow gap semiconductor physics, such as: narrow gap width formula and intrinsic absorption coefficient expression, which are very important. Of course, other results such as the expression of intrinsic carrier concentration, the expression of the effective mass of the conduction band electron, the expression of the refraction coefficient, as well as the energy band parameters, lattice vibration, impurity defects and so on are also important. Models built on these research can be used in the development of mercury cadmium telluride devices.

The second is the mercury cadmium telluride surface inversion layer Sub-band model. Two-dimensional electron gas has been a popular research area. If the theoretical calculation is carried out in the study of sub-band, the results generally differ greatly from the experimental results. I established a new model based on the Schrodinger equation, in which the sub-band energy is written into a polynomial expression of the sub-band electron concentration Ns, and then introduced a wave function release coefficient, which is combined with the Poisson equation to form a system of sub-band model equations. Several coefficients in the model can be further determined by comparing the measurement results of SdH oscillation and magneto-optical spectrum by fitting the measurement of the capacitance spectrum of the sample (CV curve). In this way, the obtained results are more in line with the actual results than those calculated theoretically. Prof. Kun Huang, the founder of semiconductor science and technology in China, spoke highly of this work, calling it a great example of China’s research achievements in two-dimensional electronic gas field.

**Figure:**
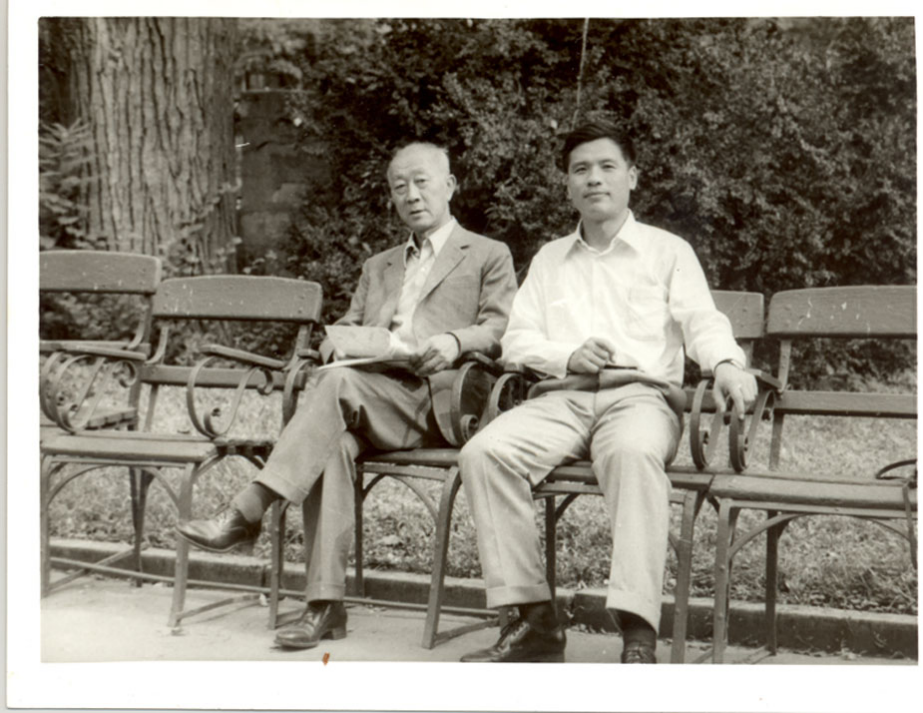
Academician Kun Huang and Academician Junhao Chu (right) at the International Conference on Semiconductor Physics in 1982

The third is a series of research achievements on ferroelectric materials infrared detectors. We studied a lot of ferroelectric materials, measured their spectra and wrote many optical parameters into the scientific manual. We also prepared ferroelectric infrared detectors to enable infrared thermal imaging.

The work continues, and in the last few years, I think two things stood out. One is that my student Zhiming Huang and his team discovered a new photoconductivity phenomenon and invented a detector that can work at room temperature in the terahertz band: the semiconductor-metal-semiconductor structure interacts with the terahertz light to induce potential well in the semiconductor material, thus binding the charge carriers from the metal and causing the conductivity to change. This discovery is of great significance to the invention of a new generation of infrared detectors, especially terahertz detectors working at room temperature. It was pioneering work.

Another important study is on the photoelectric performance of semiconductor regulated by ferroelectric polarization field, conducted by my student Jianlu Wang and his team. The polarization field of ferroelectric materials is used to regulate the behavior of semiconductor carriers and band structure, so as to effectively improve the photoelectric performance and detection rate of semiconductor and allow band regulation. They have also been carrying out cutting-edge research on two-dimensional materials used in infrared detection recently.

**3. You have long carried out research on material physics and devices of narrow gap semiconductor mercury cadmium telluride and ferroelectric thin films used in infrared detectors. Could you briefly talk about their application areas and directions?**

Academician Chu: What I have been studying is the interaction between infrared electromagnetic radiation and materials (including semiconductor or ferroelectric materials, two-dimensional materials). We work to find out how they work and use that knowledge to drive the development of materials and devices.

For example, the laws of physics which we discovered concerning narrow-gap semiconductor are not only used to guide the research of material devices to improve their performance, but also in device design, especially in the design of multi-band devices. Mercury cadmium telluride infrared detectors are widely used in the field of space science and technology all over the world.

**4. Two-dimensional materials are widely studied in the field of detectors in visible and infrared bands. Compared with traditional mercury cadmium telluride materials, how do you think two-dimensional materials will affect the application market in the future? In what special fields does it have potential?**

Academician Chu: Two-dimensional materials are attracting a lot of attention right now, with a large number of research articles published and overall development at a good level. If two-dimensional materials meet certain standards for infrared detectors, they could replace mercury cadmium telluride. As a matter of fact, mercury cadmium telluride material has certain disadvantages, because mercury is a heavy metal with very unstable chemical properties, it is difficult to prepare a large area of uniform performance with it. In addition, mercury cadmium telluride infrared detectors have to work at a temperature as low as 77 K, another disadvantage. However, at the moment due to limited development of materials studies, we are yet to find a better replacement.

Compared with traditional materials, two-dimensional materials have excellent photoelectric properties. Many scientists are trying to design new structures by coupling two-dimensional materials with other ferroelectric materials. Two-dimensional materials also have special sensitivity to polarization and unique advantages in infrared polarization measurement.

We hope that 2D materials can be used in the future to replace indium gallium arsenic at near infrared, indium antimonide, lead sulfide, and eventually mercury cadmium telluride at mid-infrared. The possibilities are there, but further research is needed. We can start with experimental and theoretical research, and then design high-performance sensors and infrared sensors when the technology is fully mature. In one sentence, two-dimensional materials have great potential.

**5. Infrared, terahertz and millimeter waves are all important optical development directions. For detection technology, multiple indicators are involved, yet there are often mutual constraints between multiple indicators. What do you think is the future of related detectors?**

Academician Chu: The development direction of the detectors is as follows:

The first is large image surface, we have to make more pixels, because the more pixels, the clearer the image. The second is wide bandwidth, wide enough to cover all bands of infrared and far infrared. It is also necessary to develop multi-band detectors such as two-color and three-color detectors, because sometimes single band information may not be accurate enough, whereas information collected in multiple bands would be helpful to analysis and judgment. The third is to raise the operating temperature of the detectors, for example, the current mercury cadmium telluride detector only works at 77 K, which is not convenient, so we want to raise the operating temperature to semiconductor refrigeration level, or ideally room temperature. The fourth is the development of terahertz infrared detector. Not long ago, Zhiming Huang, a researcher of SITP, and also my student, has successfully developed a wide band, high sensitivity, low noise equivalent power and fast response terahertz detector of 0.3–3.0 mm with narrow gap semiconductor, which proved that high sensitivity terahertz detection can be achieved through new photoelectric effect law generated by the photon wave. This work provides a new possibility for the breakthrough of terahertz detection technology. The fifth is the development of intelligent detectors that can process received information on a chip.

I think detection technology needs to meet the demand of being of high sensitivity, high detection rate and high reaction speed in any detection band. You can have one feature particularly heightened, but you have to make sure that the other features are also there. You don’t want to emphasize one at the expense of the others, because then the device would not be usable.

**6. Intensity, polarization, spectrum and phase are the four basic attributes of light waves. At present, perception of incident signals by photodetectors is gradually evolving from single intensity dimension perception to multi-dimensional collaborative perception. Could you forecast the development trend of multi-dimensional collaborative perception and the possible influence of this technology in the future?**

Academician Chu: At present, infrared detection mainly focuses on wavelength and intensity to achieve infrared imaging and temperature detection, which has been widely applied.

Developments have been made in polarization detection in recent years. Different devices have different levels of sensitivity to polarization, so polarization sensitive infrared materials and detectors need to be developed. Some ground object spectra have polarization characteristics, which can be analyzed and judged by polarization detection. In polarization related research, polarization elements such as polarizers are also very important. The Micius Satellite for Quantum Science Experiments, China’s first space quantum science experiment satellite, uses a lot of polarization elements with very high polarization ratio to determine quantum entanglement by detecting the polarization characteristics of photons. So polarization detection and polarization elements are very important.

In terms of wavelength (frequency), intensity, polarization and phase detection, the first two aspects are going well so far, polarization detection is moving in a good direction. Phase detection is also being researched. It is more challenging, but has great potential for development. There is still much to be done for researchers.

**7. You once worked on intrinsic light absorption of mercury cadmium telluride, and successfully solved the problem of high absorption coefficient measurement of mercury cadmium telluride and sample preparation. Could you tell us a little bit about your experience of grinding samples down to a few microns and discovering the first interband transition intrinsic absorption spectrum of mercury cadmium telluride?**

Academician Chu: In 1978, I became a postgraduate student under Prof. Dingyuan Tang, who set me a difficult task: find the intrinsic absorption spectrum of mercury cadmium telluride. He said no one in the world had been able to do it, and he wanted me to give it a go. I was happy to take the challenge. At that time, researchers working on the absorption spectrum of mercury cadmium telluride only measured the absorption edge (absorption coefficient less than 2000cm^−1^), that is, the electron transition from the top of the valence band to the bottom of the conduction band, but could not measure the band transition with k wave vector greater than 0 (general absorption coefficient greater than 2000cm^−1^). To make an intrinsic absorption spectrum of mercury cadmium telluride interband transition, I first had to prepare samples, which must be ground very thinly. If the sample is too thick, the wavelengths with high absorption coefficients cannot penetrated and thus cannot be measured. So the samples have to be thin and have an area of about 3 mm in diameter to be suitable for spectral measurements. This was very difficult. I designed many plans and did many experiments, and eventually produced samples of different components measuring from 2 microns to 10 microns in thickness. Then I had to find a way to measure the high absorption coefficient of mercury cadmium telluride. After much hard work, I was able to measure the (Hg1- xCdxTe) component x from 0.170 to 0.443, from 4.2 K to 300 K temperature T transition with intrinsic absorption spectrum. This is still the best research result in the world even now, and has been written into internationally respected scientific handbooks.

Starting from this research, I solved a series of problems related to the photoelectric transition, including impurity absorption spectrum, lattice vibration spectrum, mobility spectrum, two-dimensional electron gas, etc. In addition, I also put forward some expressions which can reflect the key physical properties of mercury cadmium telluride, including: The expression of gap width, intrinsic carrier concentration, absorption coefficient, refraction coefficient, cut-off wavelength. I also built the physical model of two-dimensional electron gas on the surface of mercury cadmium telluride.

**Figure:**
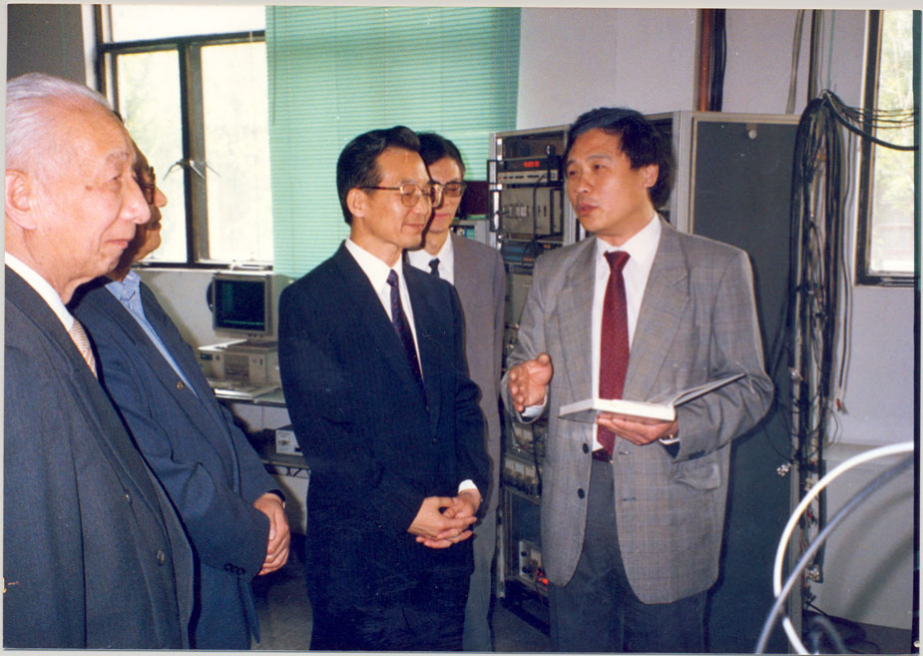
Premier Jiabao Wen visiting Academician Chu’s Laboratory

**8. Smart homes and related smart equipment are now very popular, and they would not be possible without sensors. As an expert in semiconductor physics and devices, what do you think is the future trend of smart equipment development?**

Academician Chu: Smart home is one type of applied intelligent technology, in a broader sense it is actually an intelligent system.

A good intelligent system requires three elements. First, it needs to have efficient, real-time sensors that can acquire information in real time. Second, it needs to analyze the received information just like a human brain. Third, it should respond to incoming information in a timely manner.

Without these three elements, it could only be partial intelligence, not total intelligence. So currently our smart homes are not fully intelligent, for example, if we want to turn on air conditioning before going home, we still have to use a predesigned program. Another example would be the washing machines, which are only semi-intelligent. Real intelligence would mean that when I throw a garment into it, the sensor inside can analyze what material it is made of, calculate how much detergent is needed, and determine the corresponding drying time. There are also wearable devices that collect ECG, blood pressure, heart rate, blood sugar, electroencephalogram and other information, which can be analyzed to make a diagnosis. So you see, sensors are at the core of the intelligent era, together with excellent spectral analysis and accurate control systems. The infrared detectors we work on are only one type of sensors, a small part of science. But only when all the small parts have been studied and improved can we see great development of science and technology.

**9. You have published more than 1,000 SCI journal papers and 6 monographs, edited 8 books, and holds more than 100 patents. What’s the secret of your prolificacy?**

Academician Chu: Before I was elected as an academician of CAS in 2005, I had published over 300 papers. In recent years, as I have mentored students in both East China Normal University and Fudan University, supervising their dissertation research, many students have added my name to their papers, but I generally do not like to be the corresponding author. My research team has also grown, with many young scientists flourishing rapidly and carrying out excellent research work. So the number of my publications has increased rapidly over the years.

As for books, my works include: Physics of Narrow Gap Semiconductors (Chinese and English) (Science Press, 2005), Physics and Properties of Narrow Gap Semiconductor (Springer, 2010), Devices Physics of Narrow Gap Semiconductor (Springer, 2010), Infrared Optoelectronics (Science Press, 2020), Introduction to Optoelectronic Conversion (Science Press, 2020), Perovskite-structured Iron Functional Materials (Science Press, 2022).

I worked as editor on more than 10 books, including the authoritative international science handbook “Landolt-Börnstein: Numerical Data and Functional Relationships in Science and Technology”. I was chief editor of 5 SPIE international conference proceedings.

I was also chief editor of more than a dozen books on popular science, including: “Half of the World in Darkness -- Infrared”, “One hundred thousand Whys: Energy and Environment”, “Popular Science Reader on Strategic Emerging Industries” (8 volumes), “Popular Reader on Made in China 2025” (8 volumes), “Science Starting Line” (7 volumes) and so on.

When I was 75 years old, East China Normal University Press published a book called “Flowing Shadow of Grass”, which recounts the story of my growth as well as some of my thoughts on life and work. It also includes some of my papers, and is very special to me.

**10. You have published a large number of popular science papers and works, and worked as a middle school physics teacher for ten years after graduating university. Why are you so interested in popular science education? What do you get from it?**

Academician Chu: I read a lot of popular science books and magazines when I was a child, including “Popular Science” and “Science Pictorial”. I studied in Shanghai Xuhui Middle School, and there were many books, including biographies of scientists, and so on, which ignited my interest in science.

**Figure:**
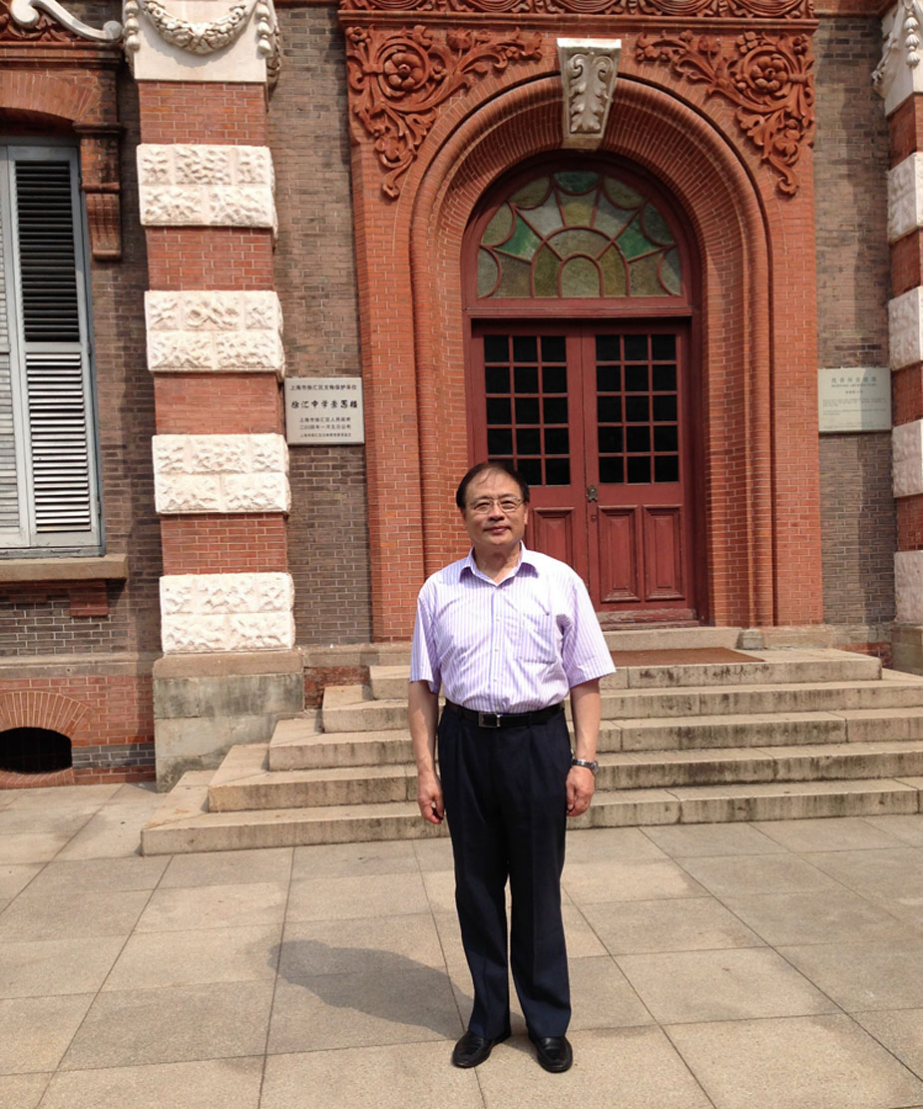
Academician Chu returns to his old high school, the Xuhui Middle School

Later, when I was a graduate student, I joined Shanghai Popular Science Creative Association, which was renamed Shanghai Popular Science Writers Association in February 2004. I published articles in the “Physics Bulletin” and “Physics Teaching” magazines.

In the past, I was busy doing scientific research, so I didn’t do a great deal of science popularization work. After 2000, I would give 2 or 3 speeches on popular science every month, always about my research area.

In addition, I have also undertaken some consulting projects as a member of faculty of CAS and made some reports, such as: “Thoughts on and Answers to the New Industrial Revolution”, “Sensor Technology in the Intelligent Age”, “Intelligent Age and Technological Innovation”, etc.

On the one hand, I have accumulated some ideas and data over the years which I would like to share. On the other hand, the feedback I got on the science popularization reports have been very good, which gives me great confidence and enthusiasm to carry on doing them.

**Figure:**
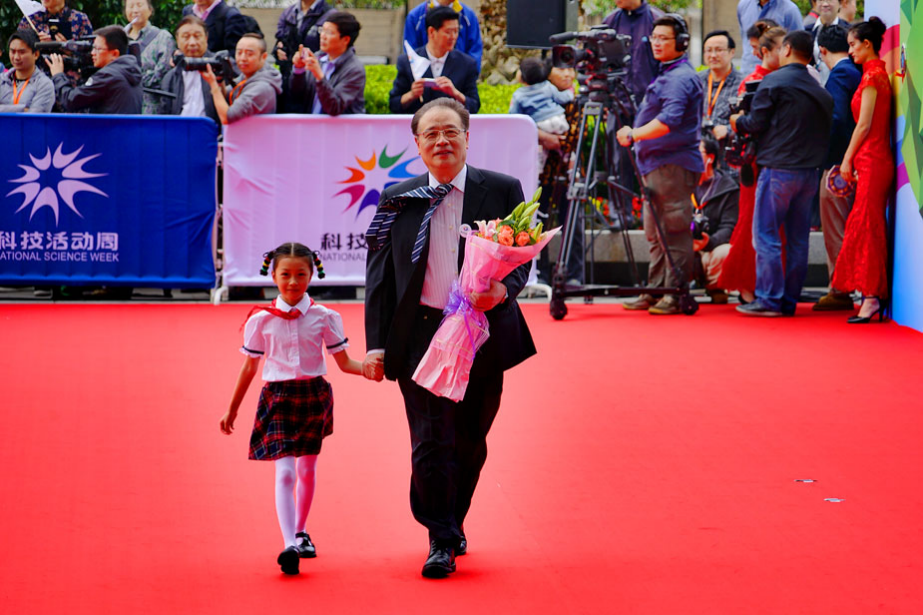
Academician Chu at the Shanghai Science and Technology Festival

**11. Your late father, Prof. Shaotang Chu, was a famous geography professor and historical geographer. He used to work in the Department of Geography of East China Normal University and was the first master’s tutor of geography education in China. What kind of influence did he have on you?**

Academician Chu: When I was a child, my father often took me to Hongkou Park (now called Lu Xun Park) near East China Normal University to teach me about astronomy, such as the science behind solar and lunar eclipses and so on.

**Figure:**
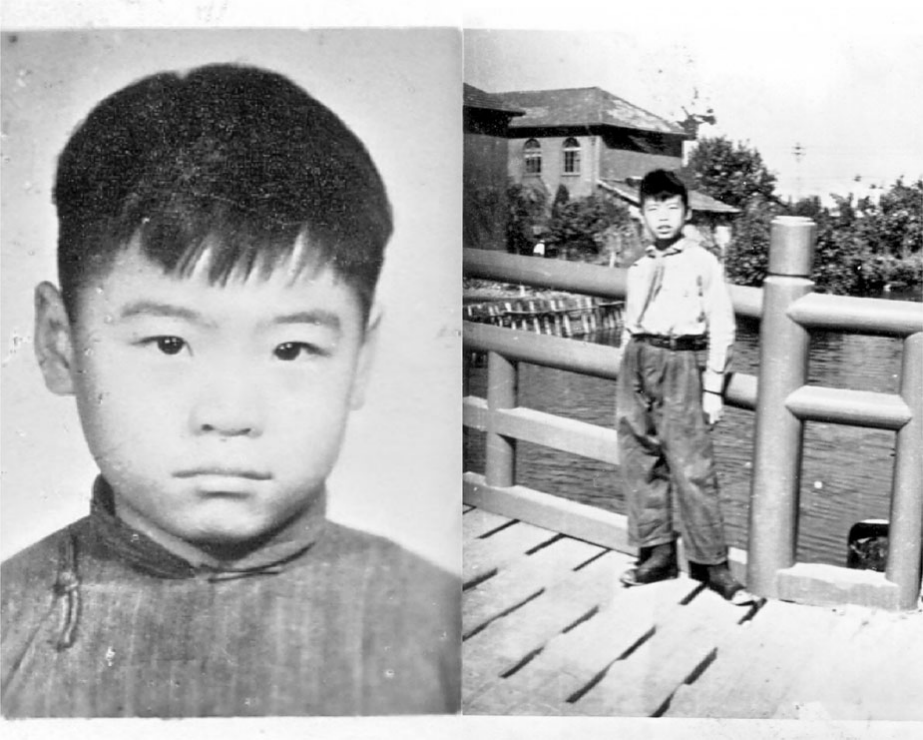
Childhood pictures of Academician Chu on the campus of East China Normal University

**Figure:**
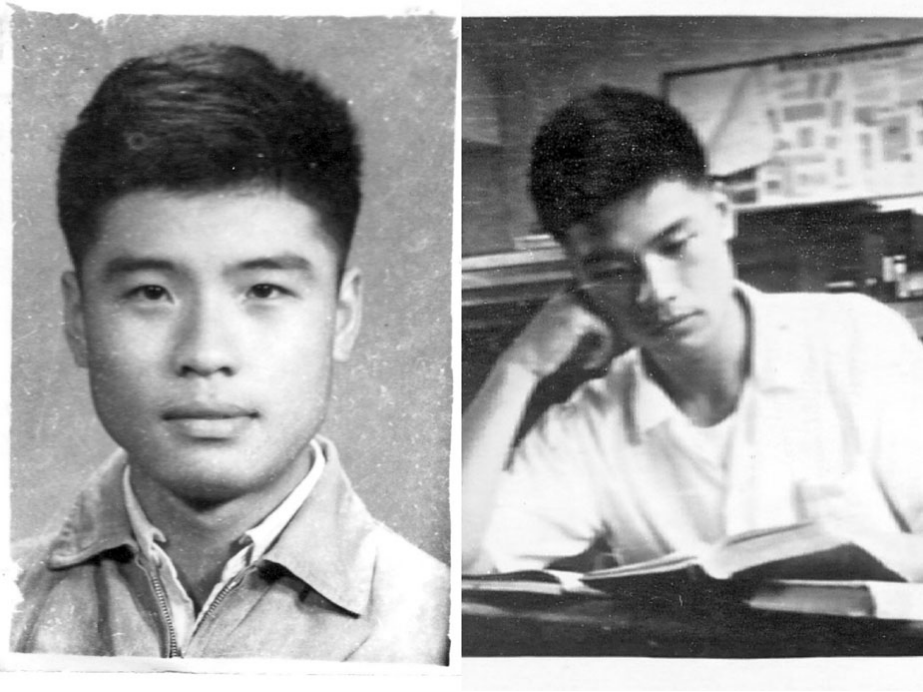
Academician Chu during his university days

My father’s influence on me is more by “example” than through “words”. My mental image of him is always of him reading or writing at his desk, or discussing geography with his students in our home. He was studious, diligent, and assiduous. He was focused in all things he undertook and loved his job. I used to tell myself that I should become a scientist just like him when I grew up.

He often told me not to be the duckweed on the surface of the pond, but to be the mullet at the bottom of the pond (that is, black fish), that means I should not seek fame or exposure, but should lay low, work hard and stay grounded.

**12. In 1986, you went to the Physics Department of the University of Munich, Germany, where you worked on semiconductor research. How did this experience influence your career?**

Academician Chu: I think this experience had a big impact on me. I went to Germany as a postdoctoral student, and carried out further study on narrow-gap semiconductor mercury-cadmium telluride two-dimensional electron gas based on the spectral study of band parameter lattice vibration of mercury-cadmium telluride. At that time, research on gallium arsenide and silicon semiconductor two-dimensional electron gas had achieved a lot of results internationally and was very advanced compared to China. Therefore, my vision was expanded at that time, especially in the experimental research methods of two-dimensional electron gas. Back then, we didn’t have any good far infrared lasers in China, nor strong magnetic field systems which could do infrared optical experiments, and many of our testing equipment were outdated. But in Germany, the laboratory had far-infrared lasers of different wavelengths, and used carbon dioxide laser to excite far-infrared gas laser. They had strong magnetic fields for electron transport testing and magneto-optical spectrum measuring, and highly sensitive capacitance measurement systems they made themselves. More importantly, I learned about the Quantum Hall Effect (QHE), a discovery which won the Nobel Prize in physics in 1985. So I think that experience helped me grow in both knowledge and experiment skills. It prepared the ground for my research on two-dimensional electron gas of narrow gap semiconductor mercury cadmium telluride, a field in which I was able to make some contributions.

During this period, I had the opportunity to talk in person to internationally respected scientists to learn how they do research and how their minds work, both of which have been very helpful for my research work. My co-supervisor in Germany at that time was Professor F. Koch, a world-class semiconductors scientist. At work, we also often held discussions with other famous scientists, including the Nobel Prize winner Klaus von Klitzing and his mentor Prof. G. Landweher and Prof. U. Roessler. In 1999, Professor Roessler, who was the editor-in-chief of the semiconductor section of the Landolt-Bornstein: Numerical Data and Functional Relationships in Science and Technology books, asked me to cover the section concerning mercury-containing compounds (including mercury cadmium telluride). I got to decide which papers on mercury-containing compounds, such as cadmium telluride, would be included in the famous scientific handbook, which was and is considered the most authoritative scientific manual in academia. The handbooks are revised every 10 years or so, and I have participated in three revisions. I think I was selected because I have done a lot of work on narrow gap semiconductor mercury cadmium telluride, but also due to a mutual understanding and trust which was built and enhanced thanks to international academic exchanges.

I believe that all scientists who went abroad for further studies at that time had similar experiences. It helped us catch up with those working on the international academic frontier. After returning to China, we all tried to pass on the scientific methodologies and ideas we learned to our students, so it played an important and positive role in promoting the development of science in China.

Two groups of people have played important roles in the development of science and technology in China. The first are scholars who returned to China from overseas when new China was first founded, and those who went abroad before the reform and opening up, such as Professors Kun Huang, Xide Xie, Dingyuan Tang, and Daheng Wang, etc. The second group is scholars who returned home after the reform and opening up. Their academic vision and scientific methods helped to promote the growth and progress of Chinese researchers, as well as China’s science and technology as a whole.

**Figure:**
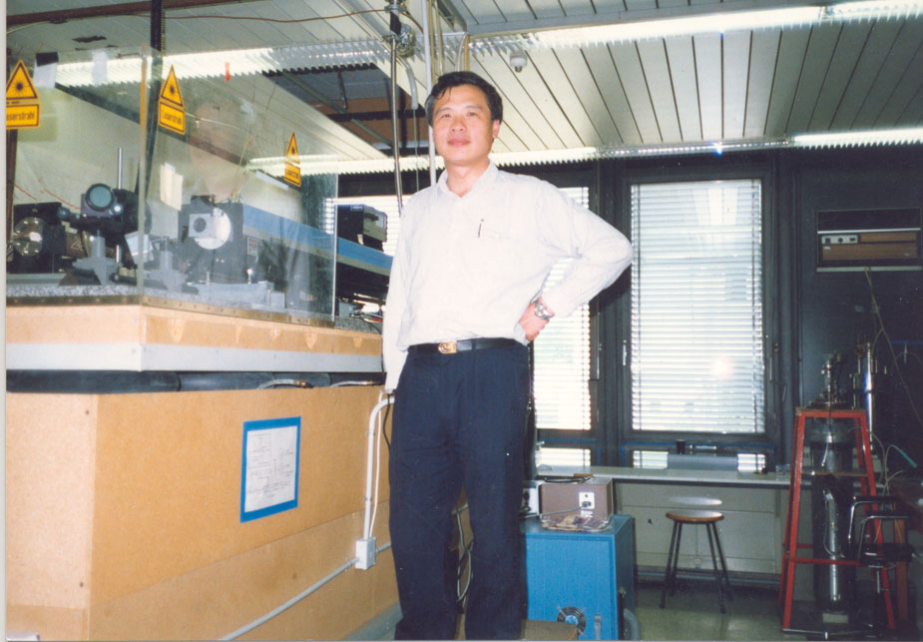
In 1986, Academician Chu in his laboratory at the Munich University of Technology

**Figure:**
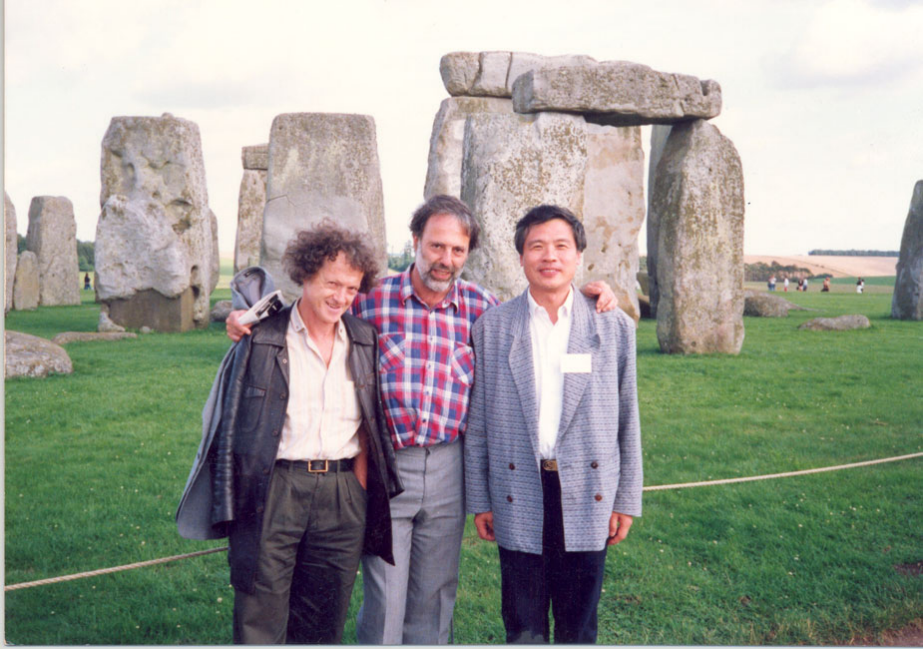
Academician Chu in with Prof. Zawaski from Poland and Prof. Pikin from Britain during the International Conference on Narrow Band Semiconductor Physics

**Figure:**
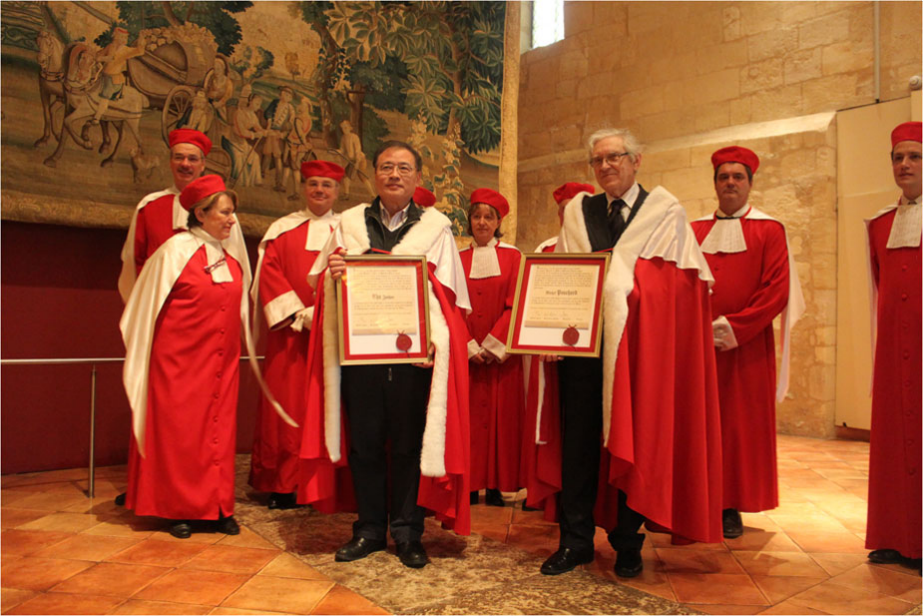
Academician Chu accepting honorary citizenship of Saint-Emilion, Bordeaux, France during the Sino-French Functional Materials Seminar

**Figure:**
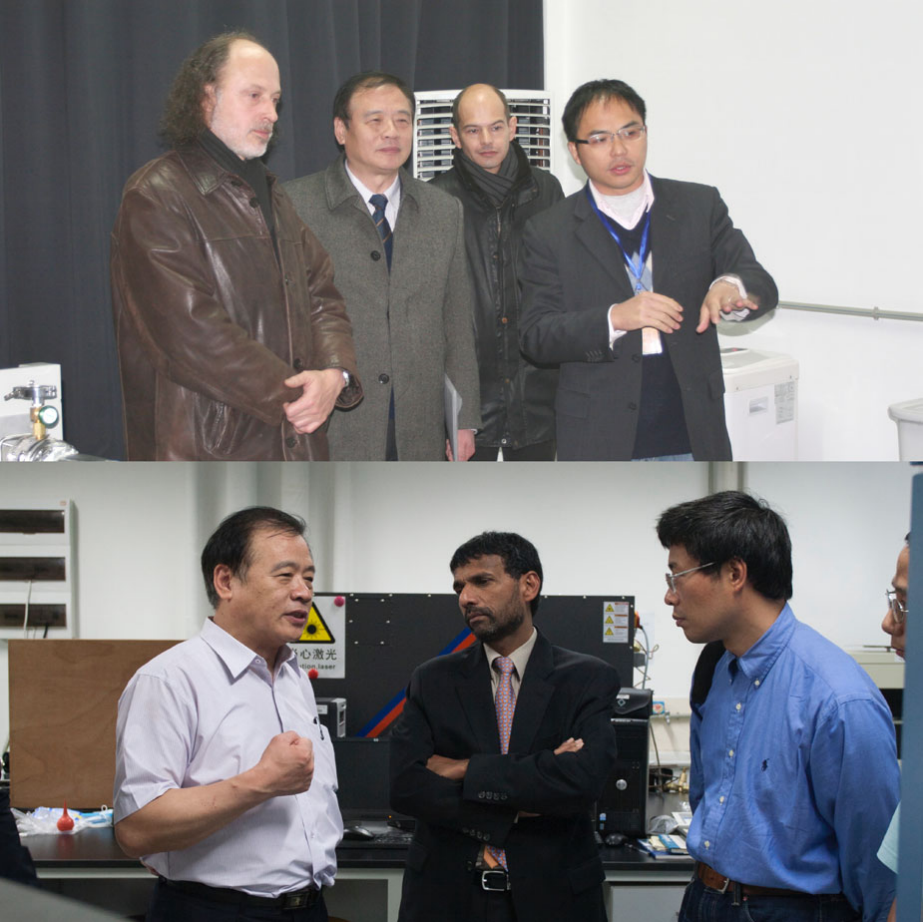
Prof. Dennis (I) from France and Prof. Siva (II) from the US on a visit to Academician Chu’s laboratory

**Figure:**
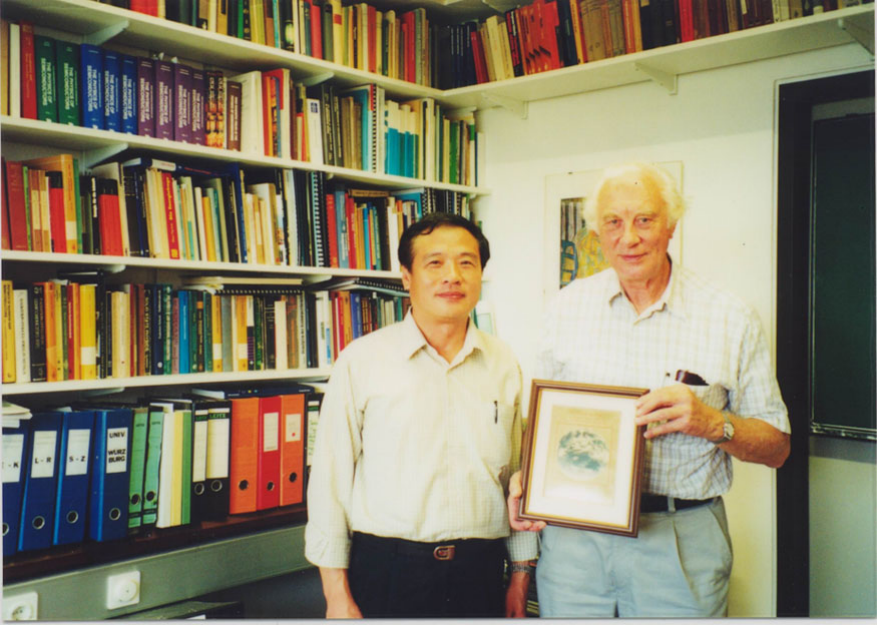
Academician Chu visiting Prof. Landwell of University of Werzburg, Germany

**Figure:**
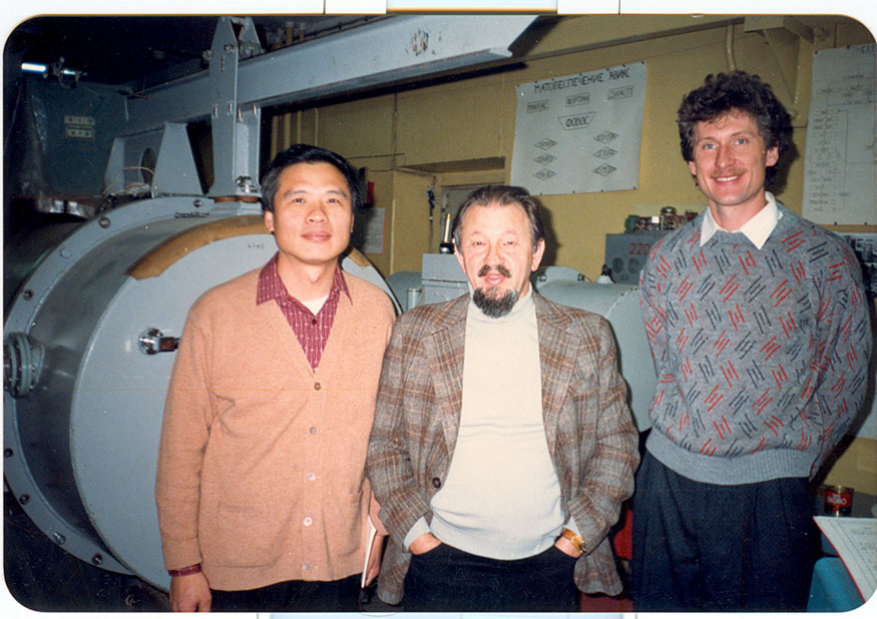
Academician Chu visiting Prof. G.N. Zhizhin at the Institute of Spectroscopy, Moscow

**13. How did you get into the study of narrow band gap semiconductor?**

Academician Chu: Even as a child, I was very interested in physics. When I graduated from university in 1966, I became a teacher in Meilong Middle School, Putuo District, Shanghai. I devoted all my spare time to physics research. When postgraduate schools were reopened in 1978, I saw my opportunity. That year, SITP recruitment exams were scheduled half a year ahead of Fudan University. Also, Prof. Dongsheng Yan, father of one of my classmates (and later CAS Vice President) suggested that I should go for SITP and took the trouble to write a recommendation letter for me. I still remember that one of the test subjects was semiconductor physics, which I never learned in university. But after six months of preparation, I got 90 out 100 in that subject, the second highest of all. I successfully became one of the first postgraduate students of SITP in 1978, and my supervisor was Prof. Dingyuan Tang, who led me into the research of narrow-gap semiconductor infrared optoelectronic physics.

**14. Professor Dingyuan Tang was one of the founders of semiconductor science and infrared science in China. What kind of influence did he have on you?**

Academician Chu: Prof. Tang, along with Professors Kun Huang, Xide Xie, and Shouwu Wang, pioneered the research of semiconductor, infrared technology and solid state physics in China.

Prof. Tang’s influence on me is mainly in two aspects. First, he was a true experimental scientist, and always stressed that we should only make experimental analysis based on actual results. Second, he told us to stay curious, and always ask questions, keep our minds focused on how to answer those questions until we could put forward our own solutions.

Prof. Tang studied in the United States, and had two papers published in *Physical Review B* (PRB) during his master’s studies. It was a highly prestigious journal, many scientists whose papers were published around the same time as Prof. Tang later went on to win Nobel Prizes. He invented a high pressure diamond vessel for optical measurement at high pressure, and discovered a new class of phase transition phenomena of the metal cerium Ce at high pressure, which was highly praised by Nobel Laureates Percy Bridgman and Pauling.

After he returned to China, Prof. Tang worked in the Institute of Physics, the Institute of Semiconductors and SITP successively. His academic spirit and attitude had a great impact on me. I still have notes I made of our discussions together.

**Figure:**
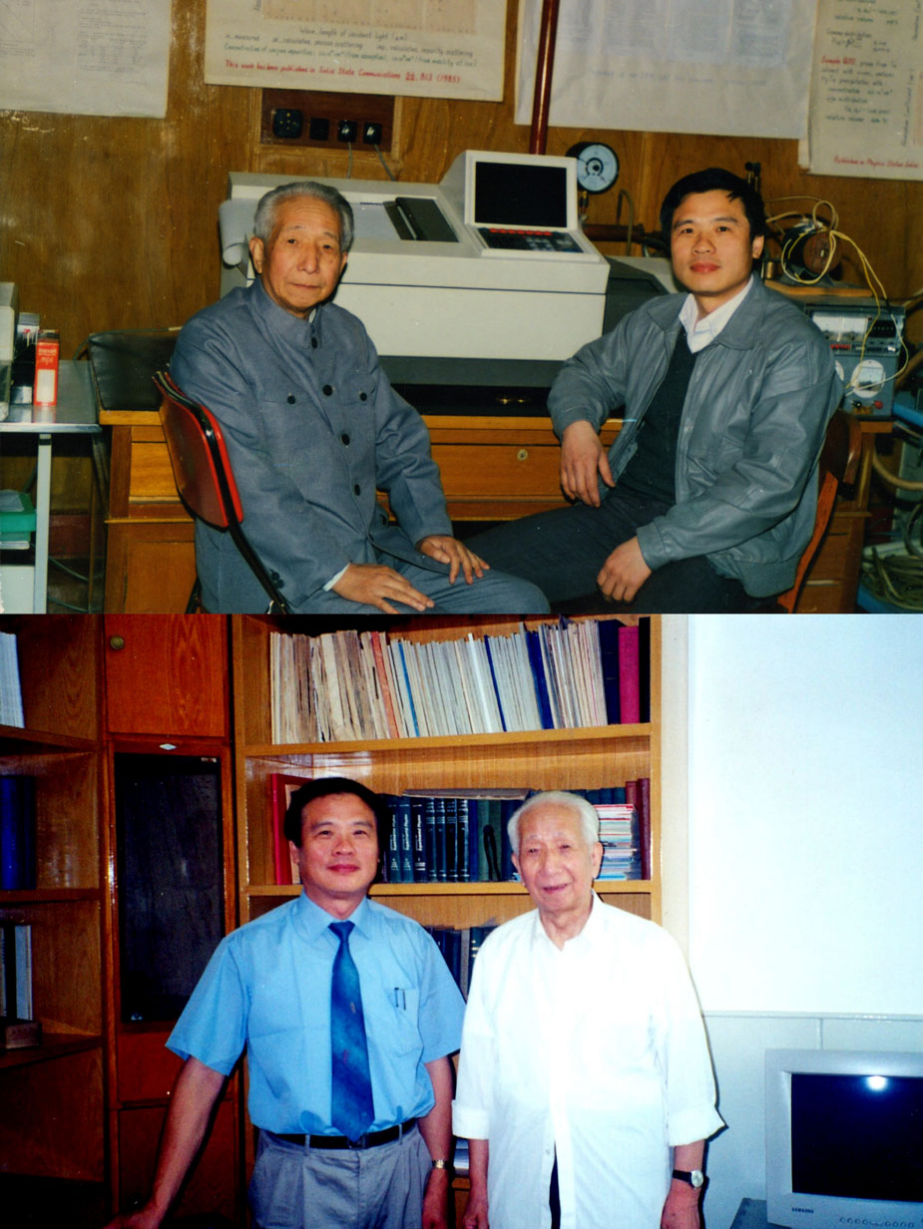
Academician Chu and his mentor Academician Dingyuan Tang

**15. As a mentor, how do you train your students?**

Academician Chu: In my opinion, students should not only be “specialists”, but also should have a “breadth” of knowledge. To qualify for a doctoral degree requires students to not only master specialized knowledge and skills in their own research area, but also related fields.

Take the spectrum experiment as an example, some students will wonder how the sample is prepared, how the material is grown, and what are its crystal structure and electrical properties. When measuring light spectrum, they’d be contemplating the structure of the spectrometer and why and how it works. Some might even open it up to see how the test process is done. I would say these are all good. However, if a student simply takes a sample to measure the spectrum, or sends it elsewhere to be measured, even though he or she may have the same measurements and can analyze the spectra, I don’t think they have received enough training. The results they get may not be comprehensive, or the analysis may not be accurate, and yet they wouldn’t know exactly what the problem is. So we train students to draw a circle around their work, to understand not only what they do, but also what is relevant to their research. Otherwise, the students will end up having narrow visions and lesser abilities.

In addition, most of the time in research, we use existing models and theories to come up with new solutions, test existing solutions to find problems in order to make improvements, or put forward new models. If you are able to do this, then you have made contribution. So in research, we must keep our minds open in order to see links between things or see into things to find the truth hidden behind the phenomenon. This is how we test and improve ourselves. In training students, we should make sure they know how to learn new knowledge, to be able to “learn by themselves”; and we want them to be able to solve actual problems. They should not only become specialists of their own fields, but also acquire a good understanding of relevant subjects. They should have both nimble minds and nimble hands, know where their work fits in the general picture of scientific research and understand the relationship and situation of the front and back-end fields which are closely related to their work.

**Figure:**
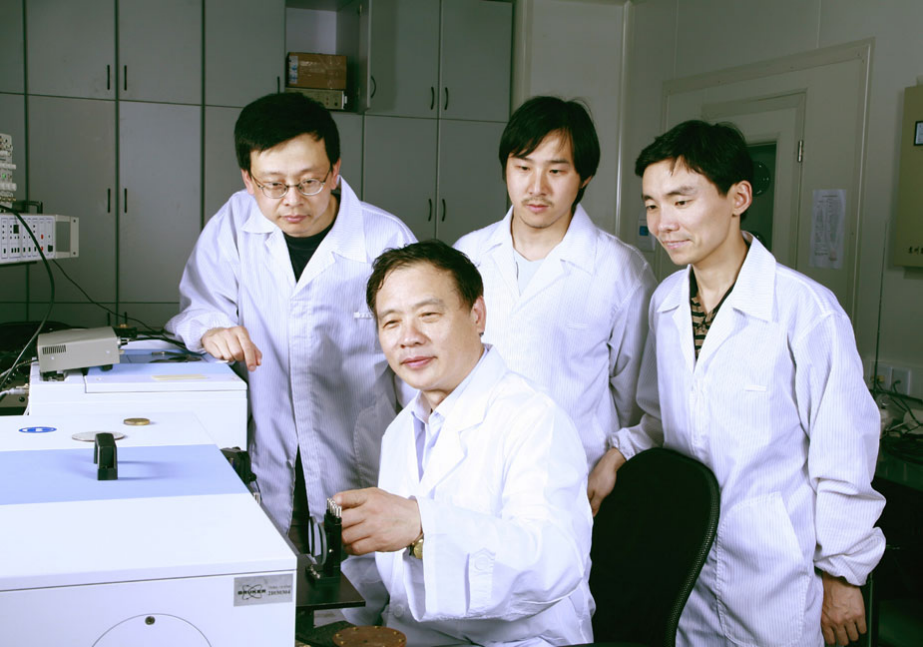
Academician Chu and his students in the spectral laboratory

**16. What qualities do you value most in a student?**

Academician Chu: Most importantly, I think students should be grounded and diligent. Secondly, they should be curious, good at asking questions, good at using their brains to solve problems. I want them to be able to do scientific research well in a down-to-earth way and not to overreach, and have the tenacity to overcome difficulties. Finally, I think they should be ambitious, and have the confidence that they can make some achievements in their fields, and make sure every little thing is done perfectly.

**17. Your team is leading in the field of cutting-edge basic research and engineering application in China, and has successfully applied the results of basic research into engineering practice. I believe that young people in your team also need to face cutting-edge basic research and engineering application research, how do you think they should balance the two?**

Academician Chu: This should be determined by their actual work. If you are in an engineering-oriented research group, then the focus should be on engineering, but even so you should be prepared to find and master the laws of physics you come across. If you are in a research group of material devices and physics, the focus will be on basic research and discovering scientific laws and mastering the technology of devices, but you still need to work on your engineering skills so you can improve the performance of materials or devices so your research could be used by industry. If you work in industry, the focus should be on how to reduce costs, improve efficiency and stabilize the process. Young people should first do their own work well, but the connections between the branches are there. For example, after doing a good job in basic research and publishing articles, they should see if they can summarize the laws of physics through solving scientific problems, build models, promote technological development and engineering application.

The Chinese philosopher Zhuang Zhou once advised, a technique would only work if it follows Tao. I think here we can say Tao can mean laws of science, laws of physics. So the lesson we take from this should be that scientific research and industry should be organically connected and mutually reinforcing. The ultimate purpose of scientific research is to discover nature’s secrets, and through industry use that knowledge to serve mankind. Industrialization promotes the continuous innovation and progress of technology. We find out laws of science through research, that lead to technological progress, and finally to industrial application.

“Learning without thinking leads to confusion, thinking without learning ends in danger,” said Confucius. In scientific research we do a lot of repetitive work, and many laws of nature are discovered through those repetitive work. We do technical work, complete engineering tasks, or industrialization work, if we simply focus on the technology itself, and never think about the “Tao (i.e. law)” behind it, then we will not be able to make progress. If we just focus on industry without thinking about the development trend of technology, we will never be able to upgrade or innovate technologies. Therefore, the relationship between the two is not contradictory, but complementary.

**18. What are your hobbies?**

Academician Chu: I used to enjoy swimming, playing badminton and volleyball, but now I like listening to music, walking and watching TV.

**19. What advice do you have for young science workers?**

Academician Chu: First of all, you should have a strong interest in scientific research and love the research work you are engaged in. Secondly, you should master scientific research methodology according to the laws of scientific development. Last but least, you should have the spirit of scientific research, never be afraid of difficulties, have perseverance and sense of responsibility.

